# Cardiovascular disease risk in patients with elevated LDL-C levels: FH vs. non-FH

**DOI:** 10.3389/fcvm.2024.1434392

**Published:** 2024-10-24

**Authors:** Haomin Huang, Lamei Li, Anni Yang, Tao Chen, Ganwei Shi, Feng Li, Luya Wang, Gaojun Cai

**Affiliations:** ^1^ Department of Cardiology, The First Affiliated Hospital of Wenzhou Medical University, Wenzhou, Zhejiang, China; ^2^ Department of Cardiology, Wujin Hospital Affiliated with Jiangsu University, The Wujin Clinical College of Xuzhou Medical University, Changzhou, Jiangsu, China; ^3^ Department of Atherosclerosis, Beijing Institute of Heart, Lung and Blood Vessel Diseases, Beijing Anzhen Hospital, Capital Medical University, Beijing, China

**Keywords:** familial hypercholesterolemia, coronary artery disease, genetic testing, inherited disease, polygenic risk score

## Abstract

**Introduction:**

Coronary artery disease (CAD) remains the primary cause of death worldwide, and familial hypercholesterolemia (FH) is a common disease that leads to CAD. This study aimed to explore the difference in CAD risk between FH and non-FH patients with high low-density lipoprotein cholesterol (LDL-C) levels.

**Methods:**

Individuals (≥18 years) who underwent coronary angiography (CAG) from June 2016 to September 2020 were consecutively enrolled. Participants with LDL-C levels ≥4.0 mmol/L were ultimately included in this study. For all participants, next-generation sequencing was performed with expanded gene panels including 11 genes (LDLR, APOB, PCSK9, LDLRAP1, ABCG5, ABCG8, LIPA, LPA, APOBR, LRPAP1, and STAP1).

**Results:**

A total of 223 individuals were included in this study. According to the CAG findings, 199 CAD patients and 24 non-CAD patients were included. The proportions of FH genes, regardless of whether 3 major genes or all 11 genes were sequenced, were not significantly different between the CAD and non-CAD groups (*P* > 0.05). In addition, all CAD patients were divided into a triple vessel disease (TVD) group and a non-TVD group. The TVD group had a greater proportion of patients with mutations in 3 FH major genes (*P* < 0.05). In addition, TC, LDL-C and modified LDL-C (MLDL-C) levels were higher and the estimated glomerular filtration rate (eGFR) was lower in the TVD group than in the non-TVD group (all *P* < 0.05). However, multivariate logistic regression analyses revealed that only the eGFR was an independent risk factor for TVD (OR 0.99; 95% CI: 0.98–1.00, *P* < 0.05). To eliminate the impact of the eGFR, subgroup analysis was conducted, and the results indicated that among CAD patients in the high-eGFR group, having FH mutations in 3 major genes was an independent risk factor for TVD (OR 3.00; 95% CI: 1.16–7.79, *P* < 0.05). In total, 104 FH-related mutations were detected in this study.

**Conclusions:**

FH mutation did not increase the rate of CAD in individuals with an MLDL-C level ≥4.0 mmol/L. However, among CAD patients (MLDL-C level ≥4.0 mmol/L) with almost normal renal function (≥87.4 ml/min/1.73 m^2^), the probability of enduring TVD in those with FH mutations in 3 major genes was 3.00 times greater than that in those without FH mutations.

## Introduction

Coronary artery disease (CAD) remains one of the most common causes of death worldwide (1). There are approximately 11.4 million CAD patients in China (2). Dyslipidemia, especially elevated low-density lipoprotein cholesterol (LDL-C) levels, plays an important role in and significantly increases the risk of CAD. In addition to acquired causes such as unhealthy dietary habits and lack of exercise, familial hypercholesterolemia (FH, OMIM#143890), an autosomal dominant inherited disease, markedly increases LDL-C levels.

FH is characterized by high LDL-C levels, tendon xanthomas, corneal arcus (<45 years), and premature coronary artery disease (PCAD) (3). A recent meta-analysis showed that approximately 1 in 311 individuals in the general population were affected by FH, and the pooled prevalence of FH among those with CAD was 18-fold greater than that of the general population (4). Continued increases in LDL-C levels strongly increase the risk of cardiovascular disease (CVD). Without early diagnosis and appropriate treatment, FH individuals usually have a dramatically shortened life expectancy, especially for individuals with homozygous FH and fatal cardiovascular complications in childhood (5).

Like other inherited diseases, FH is diagnosed via clinical phenotype and molecular genotype. To date, there are various clinical diagnostic criteria for FH, including the Dutch Lipid Clinical Network (DLCN) criteria (6), the Make Early Diagnosis to Prevent Early Death criteria (7), the Simon-Broome diagnostic criteria (8) and the Modified DLCN criteria (9). However, the international expert panel suggested that genetic testing should still be regarded as the “gold standard” for FH (10). As a previous study reported, the causative genes of most FH cases are the low-density lipoprotein receptor (LDLR), apolipoprotein B (APOB), and proprotein convertase subtilisin/kexin type 9 (PCSK9) genes (11). However, some patients with a phenotypic diagnosis of FH do not harbor variants in these three major risk genes (12). These phenomena and research on the molecular genetics of dyslipidemias suggest that the inheritance patterns of FH might be more complex than previously thought and that there are other “minor FH genes” in addition to these three major genes. Variants in these genes are less common but might also cause an FH phenotype (13). Cao et al. (14, 15) suggested that expanding genetic testing may further elucidate the causes of phenotypic FH. As a result, expanded gene panels need to be generated to improve the diagnostic rate of FH in clinical practice (10).

A previous study demonstrated that patients with FH have a markedly increased risk for CVD compared with the general population due to high LDL-C levels (16). Among those individuals with elevated LDL-C levels, some had FH and harbored related mutations. Some non-FH individuals had other inherited diseases, such as dominant dysbetalipoproteinemia, sitosterolemia and lysosomal acid lipase deficiency; alternatively, some individuals had poor living habits, such as a high-fat diet and a lack of exercise (17). However, is the risk of suffering CAD different between individuals with FH and those without FH in a cohort of high LDL-C individuals? Do those patients with FH have more severe CAD and require more attention? These questions are related to clinical practice and therapeutic regimens. At present, few studies have investigated these two questions using next-generation sequencing (NGS) via an expanded gene panel. Thus, the present study aimed to explore the difference in CAD risk between FH and non-FH patients with high LDL-C levels via an expanded gene panel via NGS. And in view of CAD is a highly heritable trait related to LDL-C concentrations, LDLC-affecting Polygenic Risk Scores (PRS) is also conducted to predict PCAD in this study.

## Methods

### Participants

A total of 4,165 individuals (≥18 years) who underwent coronary angiography (CAG) between June 2016 and September 2020 were enrolled in the study. Patients with liver dysfunction, serious renal insufficiency, hypothyroidism, or a history of cancer or those without blood samples were excluded. After the exclusion criteria were met, a total of 223 individuals with modified LDL-C (MLDL-C) levels ≥4.0 mmol/L were ultimately enrolled in this study.

This study was conducted in accordance with the Declaration of Helsinki and was approved by the Institutional Ethics Committee of Wujin Hospital (Ethics approval number: 201606). Written informed consent was obtained from all participants.

### Data collection

The clinical data of all the participants, which included sex, age, height, weight, systolic blood pressure (SBP), diastolic blood pressure (DBP), heart rate (HR), smoking status, drinking status, primary hypertension (PH) status, type 2 diabetes mellitus (T2DM) status, CAD status and the type of lipid-lowering drugs used, were acquired from electronic records.

Venous blood samples were collected from all participants after they had fasted for at least 12 h. The blood cells were then extracted by two professional workers and preserved at −80°C. Biochemical parameters, including platelet (PLT), white blood cell (WBC), alanine transaminase (ALT), aspartate aminotransferase (AST), total bilirubin (TBIL), creatinine (Cr), total cholesterol (TC), triglyceride (TG), high-density lipoprotein cholesterol (HDL-C), and LDL-C levels, were measured via an Beckman AU5800 automated biochemical analyzer. Among then, TC and TG were tested using BECKMAN COULTER test kit, and HDL-C and LDL-C were tested using ORIENTER test kit.

### Diagnostic criteria

Individuals with mutations in one of 3 major FH genes were identified as FH, and those with mutations in only 8 minor genes were identified as possible FH. PH was defined as a repeated SBP ≥140 mmHg and/or DBP ≥90 mmHg at least three times on different days and without secondary factors. T2DM was defined as a fasting serum glucose level ≥7.0 mmol/L and/or random glucose level ≥11.1 mmol/L plus symptoms and no secondary factors. CAD patients were diagnosed if at least one main coronary artery exhibited ≥50% stenosis. PCAD patients were diagnosed if males were identified as CAD for the first time before 55 years, and femals were before 60 years. Body mass index (BMI) was calculated as the quotient of weight divided by the square of height (weight/height^2^). Triple vessel disease (TVD) was identified as the left main coronary artery, with the right coronary artery exhibiting ≥50% stenosis or three main coronary arteries exhibiting ≥50% stenosis. Liver dysfunction was defined as ALT ≥200 U/L, serious renal insufficiency was defined as an estimated glomerular filtration rate (eGFR) < 30 ml/min/1.73 m^2^, and the formula of eGFR for males was [140 − age(year)]*weight(kg)/[Scr(mg/dl)*72], which should be multiplied by 0.85 for females.

When patients used lipid-lowering drugs before admission, their LDL-C levels were eventually multiplied by the corresponding coefficient to determine the adjusted MLDL-C level (18). Smoking and drinking were defined as described in a previous study (19).

### Genetic testing

Blood cell samples were retrieved from −80°C freezers and sent for genomic deoxyribonucleic acid (DNA) extraction following the manufacturer's standard procedure using a DNeasy Blood & Tissue Kit (QIAGEN, Hilden, Germany). DNA purity was tested via an Invitrogen Qbit Spectrophotometer. Afterward, qualified samples were selected for targeted NGS covering all the coding exons of the LDLR, APOB, PCSK9, LDLRAP1, ABCG5, ABCG8, LIPA, LPA, APOBR, LRPAP1, and STAP1 genes. The amplification reactions were carried out on an AB 2,720 Thermal Cycler (Life Technologies Corporation, USA). After cluster generation and hybridization of the sequencing primers, base incorporation was performed on a NovaSeq Benchtop Sequencer (Illumina, Inc., San Diego, CA).

### Bioinformatics analysis

Sequencing reads were aligned to hg38 using the Burrows–Wheeler Aligner. Single nucleotide variant (SNV) calling was performed via both the Genome Analysis Toolkit and Var Scan programs, and the resulting SNV data were subsequently combined. The ANNOVAR program was used for SNV annotation. The functional effect of nonsynonymous SNVs was assessed via PolyPhen-2, SIFT, and Mutation Taster. Nonsynonymous SNVs with a SIFT score <0.05, a Polyphen-2 score >0.85 or a Mutation Taster score >0.85 were considered significant and not benign. To sort potentially deleterious variants from benign polymorphisms, Perl scripts were used to filter the SNVs against those of dbSNP135. Any SNV recorded in dbSNP135 with a minor allele frequency of ≥1% in Chinese individuals from the 1,000 Genomes database was considered a benign polymorphism and therefore removed for subsequent analysis.

### Statistical analysis

SPSS 22.0 software was used to analyze the data in this study. The normality of the data was evaluated via the Kolmogorov‒Smirnov test. Continuous variables with a normal distribution are presented as the means ± standard deviations and were compared via Student's *t*-tests. Otherwise, continuous variables are presented as medians (Q1‒Q3 quartiles) and compared via the Mann‒Whitney *U*-test. Categorical variables are presented as numbers (percentages) and were estimated via the chi-square test or Fisher's test. A test for linear trends was used to determine whether there was a trend of linear change. The relationship between FH mutation and TVD was explored via binary logistic regression analyses expressed as odds ratios (ORs) with 95% confidence intervals (95% CIs). PRS for each individual were generated using PRSice-2. A two-sided *P* value <0.05 indicated statistical significance.

## Results

### Clinical and phenotypic data of the participants

As shown in Table 1, a total of 223 individuals with an average age of 58 years were ultimately included. According to the CAG findings, 199 CAD patients and 24 non-CAD patients were included. The CAD group had higher WBC counts (*P* < 0.05), but other clinical and phenotypic data, including FH mutation data, were not significantly different. Furthermore, CAD patients were divided into a TVD group and a non-TVD group. The TVD group had a greater proportion of patients with mutations in 3 FH major genes (*P* < 0.05). In addition, TC, LDL-C, and MLDL-C levels were higher and the eGFR was lower in the TVD group than in the non-TVD group (all *P* < 0.05). Other characteristics, such as sex, age, BMI, SBP, DBP, HR, T2DM, PLT, WBC, ALT, AST, and TBIL, were not significantly different between these two groups (all *P* > 0.05).

**Table 1:** Clinical and phenotypic data of whole participants.

Characteristics	Non-CAD (*n* = 24)	CAD (*n* = 199)	*P*	CAD (*n* = 199)		*P*
				Non-TVD (*n* = 120)	TVD (*n* = 79)	
Clinical data						
Male, *n* (%)	10.0 (41.7%)	120.0 (69.3%)	0.08	75.0 (62.5%)	45.0 (57.0%)	0.435
Age, year	61.5 (51.8, 71.0)	61.0 (54.0, 69.0)	0.860	59.5 (52.0, 68.0)	64.0 (55.0, 71.0)	0.092
BMI, kg/m^2^	25.2 (23.1, 27.6)	24.8 (22.7, 27.1)	0.529	24.8 (22.5, 27.0)	24.7 (22.8, 27.2)	0.192
SBP, mmHg	142.0 (125.0, 153.3)	140.0 (126.0, 153.0)	0.999	140.0 (124.3, 153.0)	140.0 (127.0, 156.0)	0.086
DBP, mmHg	80.5 (75.0, 93.5)	82.0 (76.0, 90.0)	0.583	82.5 (76.0,90.0)	82.0 (78.0, 90.0)	0.208
HR, BPM	70.5 (68.0, 80.0)	72.0 (68.0, 80.0)	0.618	72.0 (68.3, 80.0)	72.0 (68.0, 80.0)	0.062
Smoker, *n* (%)	7.0 (29.2%)	72.0 (36.2%)	0.497	44.0 (36.7%)	28.0 (35.4%)	0.860
Drinker, *n* (%)	3.0 (12.5%)	22.0 (11.1%)	0.832	15.0 (12.5%)	7.0 (8.9%)	0.423
PH, *n* (%)	13.0 (54.2%)	140.0 (70.4%)	0.107	81.0 (67.5%)	59.0 (74.7%)	0.278
T2DM, *n* (%)	4.0 (16.7%)	66.0 (33.2%)	0.100	35.0 (29.2%)	31.0 (39.2%)	0.140
Laboratory parameters						
PLT, 10^9^/L	191 (163.3, 254.0)	221.0 (180.0, 262.0)	0.199	223.5 (181.0, 262.8)	218.0 (175.0, 257.0)	0.611
WBC, 10^9^/L	5.9 (5.1, 6.7)	6.7 (5.5, 7.9)	0.020	6.8 (5.5, 8.1)	6.5 (5.4, 7.8)	0.570
ALT, U/L	23.0 (18.3, 33.5)	22.0 (15.0, 33.0)	0.337	24.0 (16.0, 36.0)	20.0 (14.0, 32.0)	0.086
AST, U/L	22.5 (20.0, 34.0)	23.0 (19.0, 33.0)	0.980	24.0 (19.0, 34.8)	23.0 (19.0, 29.0)	0.196
TBIL, μmol/L	13.4 (10.2, 17.4)	13.2 (10.4, 16.9)	0.975	13.8 (10.8, 17.0)	12.4 (10.3, 16.9)	0.914
eGFR, ml/min/1.73 m^2^	99.2 (62.5, 114.9)	87.4 (65.2, 107.1)	0.362	93.2 (71.4, 113.6)	79.4 (59.9, 102.0)	0.031
TC, mmol/L	5.6 (5.2, 6.9)	6.0 (5.1, 6.8)	0.889	5.8 (5.1, 6.7)	6.2 (4.9, 7.1)	0.002
TG, mmol/L	2.0 (1.5, 2.4)	1.8 (1.4, 2.6)	0.813	2.0 (1.4, 2.7)	1.7 (1.3, 2.4)	0.265
HDL-C, mmol/L	1.2 (1.0, 1.4)	1.1 (1.0, 1.3)	0.263	1.1 (1.1, 1.3)	1.1 (1.0, 1.3)	0.802
LDL-C, mmol/L	4.1 (3.4, 4.7)	4.1 (3.4, 4.8)	0.860	4.1 (3.4, 4.7)	4.6 (3.4, 5.1)	<0.001
MLDL-C, mmol/L	5.1 (4.7, 5.5)	5.0 (4.6, 5.8)	0.601	4.9 (4.5, 5.7)	5.1 (4.7, 6.0)	<0.001
With mutation in 3 FH major genes	5.0 (20.8%)	54.0 (27.1%)	0.508	26.0 (21.7%)	28 (35.4%)	0.032
With mutation in 11 FH related genes	11.0 (45.8%)	95 (47.7%)	0.860	53.0 (44.2%)	42.0 (53.2%)	0.214

FH, familial hypercholesterolemia; BMI, body mass index; SBP, systolic blood pressure; DBP, diastolic blood pressure; HR, heart rate; PH, primary hypertension; T2DM, type 2 diabetes mellitus; PLT, platelet; WBC, white blood cell; ALT, alanine transaminase; AST, aspartate aminotransferase; TBIL, total bilirubin; SUA, serum uric acid; eGFR, estimated glomerular filtration rate; TC, total cholesterol; TG, triglycerides; HDL-C, high-density lipoprotein cholesterol; LDL-C, low-density lipoprotein cholesterol; MLDL-C, modified LDL-C; CAD, coronary artery disease; TVD, three vessel disease.

### Frequencies of FHs and possible FHs in different MLDL-C ranges

**Figure 1:**
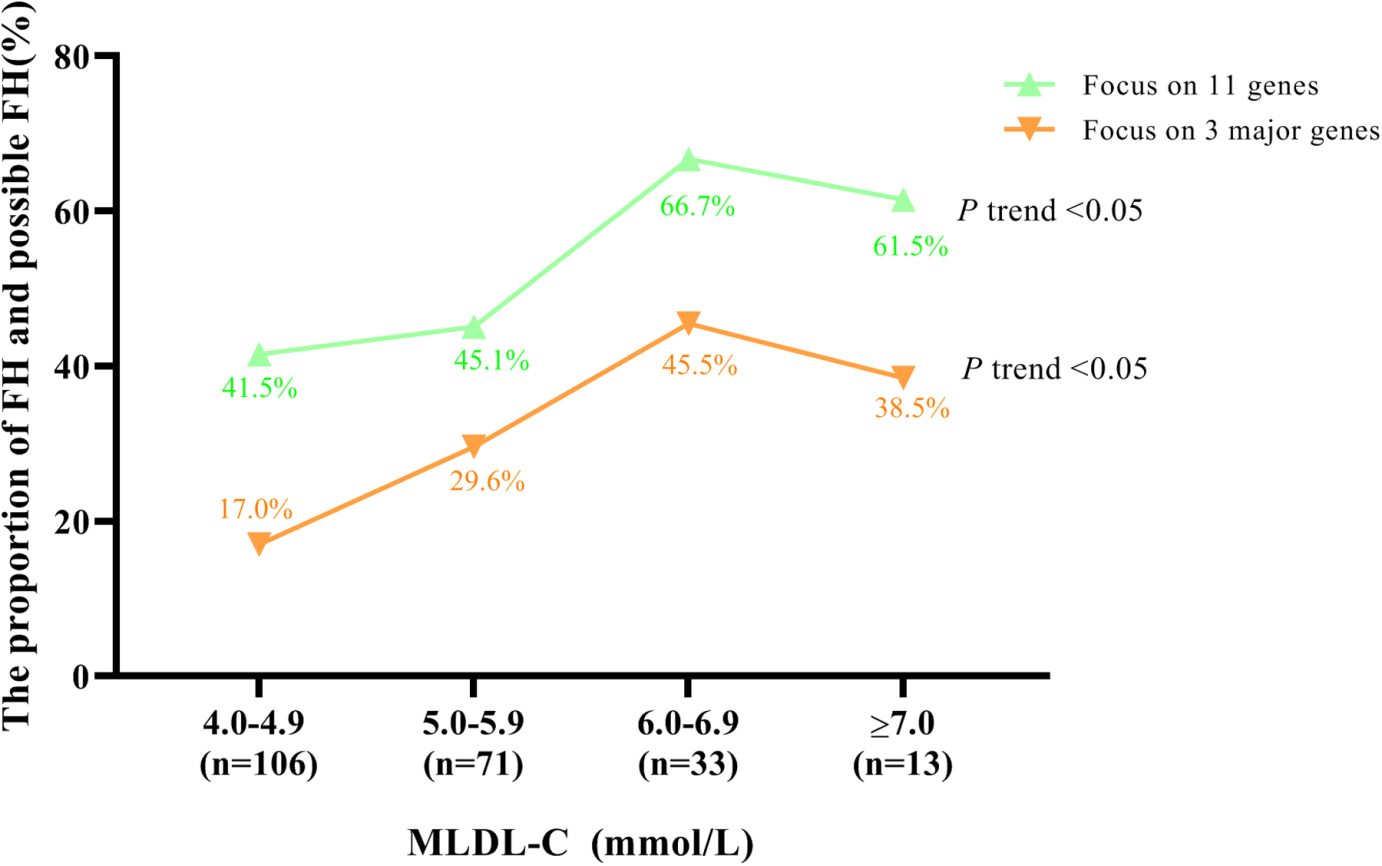
Proportion of FH patients and possible FH patients in different MLDL-C ranges. Diagram displaying the distributions of FH patients or possible FH patients by sequencing 11 FH-related genes and 3 major genes, respectively.

To explore the distributions of FH and possible FH, which were sequenced by all 11 genes and 3 major genes, in different MLDL-C ranges, all individuals were divided into 4 groups according to their MLDL-C levels, as shown in Figure 1. Generally, the frequency of FH and possible FH increased as MLDL-C levels increased, regardless of whether 11 genes or the 3 major genes were involved (*P* trend < 0.05). This frequency was relatively high, as it was 66.7% and 45.5% for MLDL-C concentrations between 6.0 and 6.9 mmol/L when 11 genes and 3 major genes were involved, respectively.

### Frequencies of FH genes in the total population

A total of 104 FH-related mutations were detected in 11 genes in this study. While sequencing for 3 major genes, 59 individuals were found to have FH mutations. Among them, 41 individuals harbored FH mutations in the LDLR, accounting for 69.5% (41/59) of the population. And 21 individuals carried FH mutations in APOB, accounting for 35.6% (21/59). The proportion of PCSK9-positive cells was 3.4% (2/59). While 11 genes were sequenced, 106 individuals were found to have FH-related mutations. Among those 8 minor genes, the LPA gene was the most common, with 32 individuals having mutations, accounting for 30.2% (32/106). And 18 individuals had mutations in ABCG5, accounting for 17% (18/106). The details of the mutations in other genes are shown in Supplementary Table S1.

### Influence of FH mutations on MLDL-C levels

**Figure 2:**
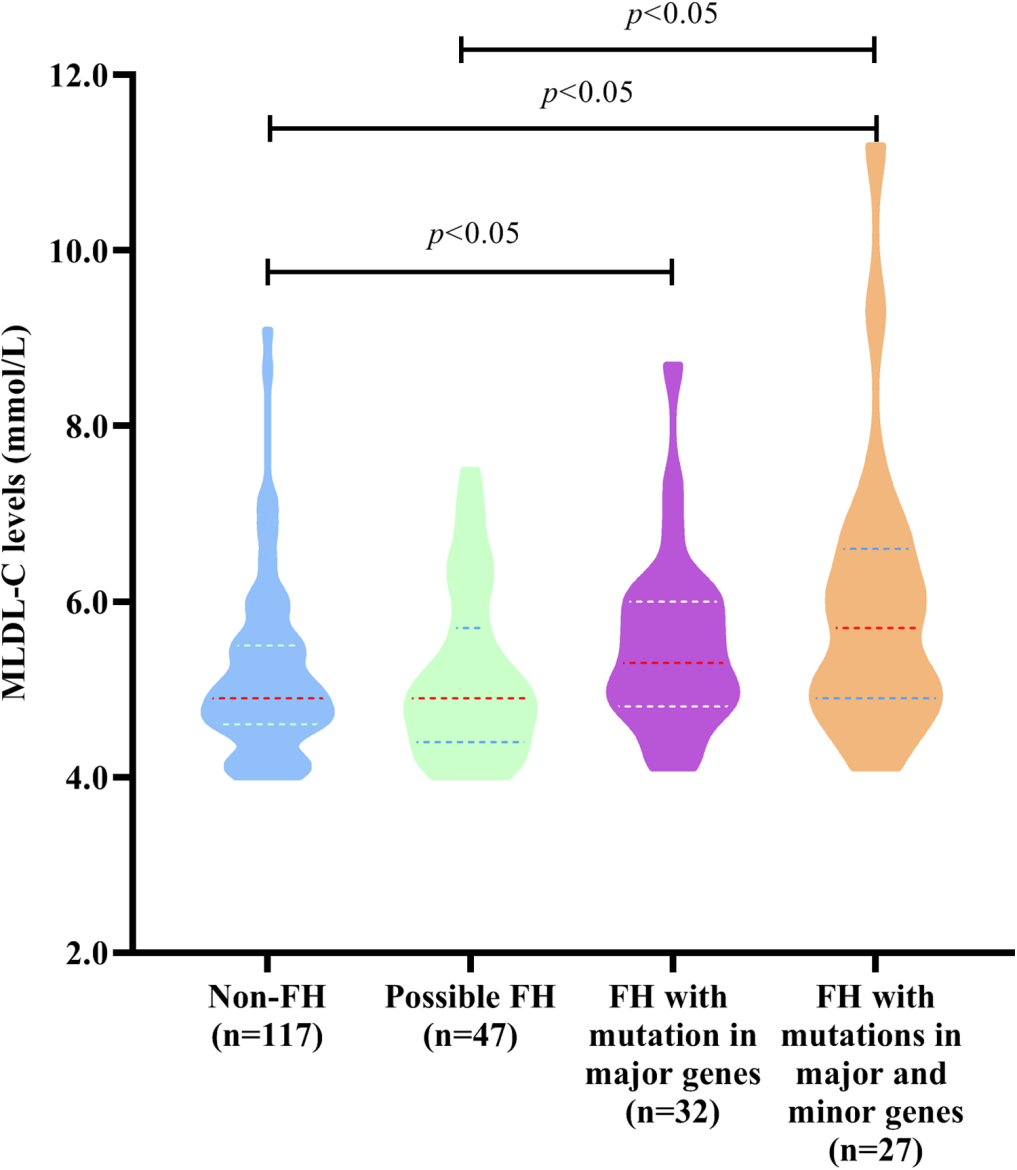
MLDL-C levels in individuals with different types of FH-related genes. Diagram displaying the MLDL-C levels in non-FH patients, possibly FH patients with mutations in only 8 minor genes, FH patients with mutations in only 3 major genes, and FH patients with mutations in both 3 major genes and 8 minor genes.

To explore whether FH mutations in major and minor genes had significantly different impacts on MLDL-C levels, all individuals were divided into 4 groups, namely, non-FH, possible FH with only minor genes, FH with only major genes, and FH with both major and minor genes, as shown in Figure 2. The MLDL-C levels in the FH group with both major and minor genes were significantly greater than those in the non-FH and possibly FH with only minor gene groups (*P* < 0.05). And it was also significantly greater in the FH group with only major genes than that in the non-FH group (*P* < 0.05).

### Influence of FH mutations on the severity of CVD

**Figure 3:**
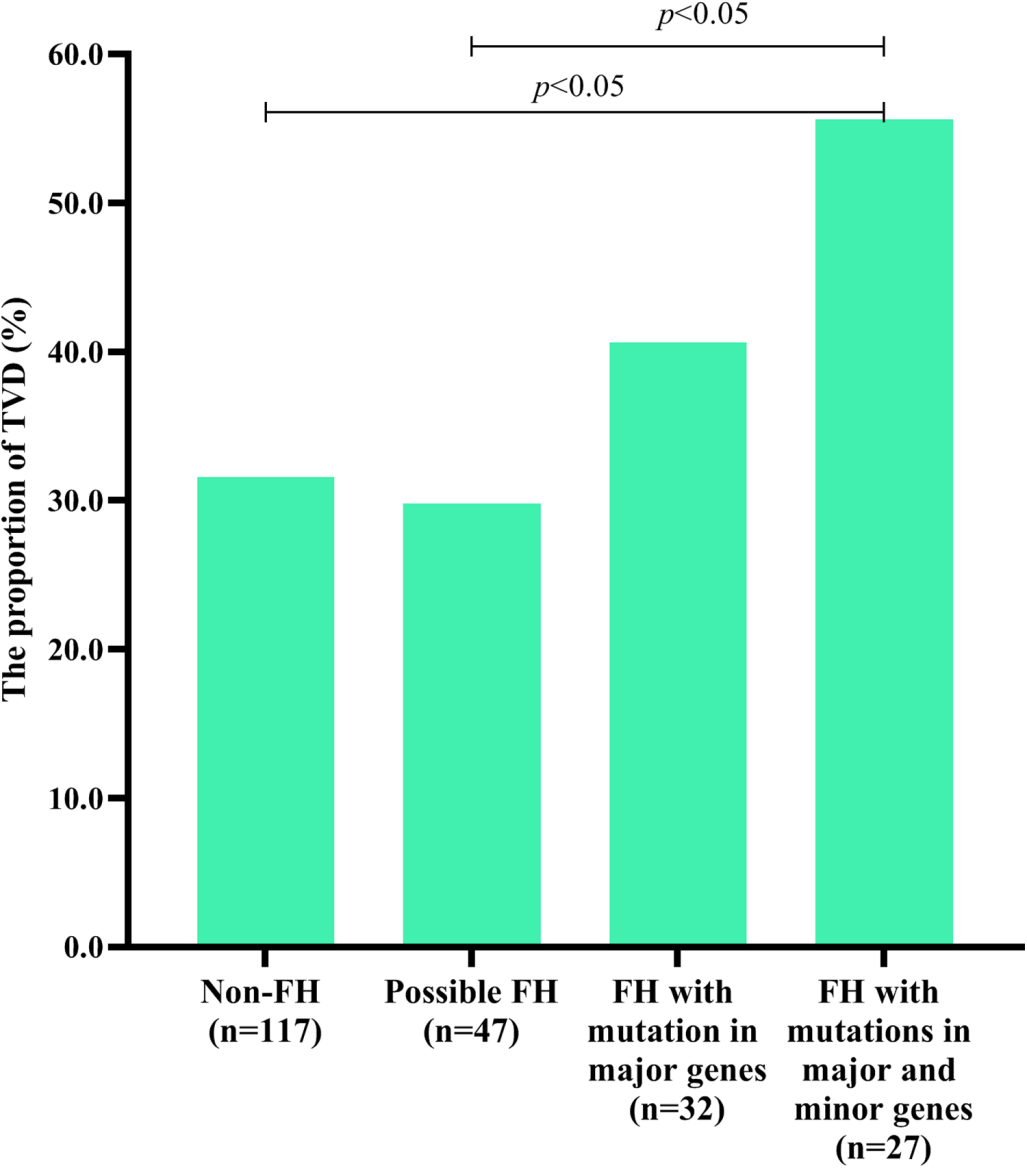
TVD in individuals with different types of FH-related genes. Diagram displaying the TVD distributions in non-FH patients, possible FH patients with mutations in only 8 minor genes, FH patients with mutations in only 3 major genes, and FH patients with mutations in both 3 major genes and 8 minor genes.

Table 1 shows that the TVD group had a greater proportion of patients with mutations in 3 major genes (*P* < 0.05). To further explore the influence of FH mutation on the severity of CVD, all individuals were divided into 4 groups. As shown in Figure 3, FH with mutations in both major and minor genes had a significantly greater proportion of TVD than did non-FH and possible FH (FH with mutations in both major and minor genes vs. non-FH, 55.6% vs. 31.6%, *P <* 0.05; FH with mutations in both major and minor genes vs. Possible FH, 55.6% vs. 29.8%, *P <* 0.05). The TVD distributions did not significantly differ between any of the other two groups (all *P* > 0.05).

### Multivariate logistic regression analyses

**Figure 4:**
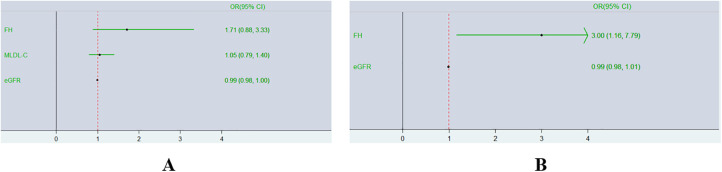
Forest plot of multivariate logistic regression analyses of TVD risk factors. Diagram outlining the relationship between having FH mutations in 3 major genes and TVD in two different groups expressed as ORs (95% CIs). (**A**),Overall CAD patients; (**B**) CAD patients with higher eGFRs.

Binary logistic regression analyses were executed in all CAD patients to explore the risk factors for TVD. As represented in Figure 4A, a multivariate logistic regression model including MLDL-C and the eGFR indicated that only the eGFR was an independent risk factor for TVD (OR 0.99; 95% CI: 0.98–1.00, *P* < 0.05). To exclude the influence of the eGFR, subgroup analyses were conducted as shown in Table 2. CAD patients were divided into two groups according to the median eGFR. Among CAD patients in the high-eGFR group, TVD patients had a greater proportion of mutations in 3 major genes than non-TVD patients did (*P* < 0.05), whereas other characteristics, such as the eGFR and/or other lipid parameters, did not differ between these two groups (all *P* > 0.05). The multivariate logistic regression analyses shown in Figure 4B revealed that having FH mutations in 3 major genes was an independent risk factor for TVD in CAD patients with higher eGFRs (OR 3.00; 95% CI: 1.16–7.79, *P* < 0.05).

**Table 2:** Clinical and phenotypic data in subgroups divided by eGFR level.

Characteristics	CAD patients with Lower eGFR (*n* = 99)			CAD patients with Higher eGFR (*n* = 100)		
	Non-TVD (*n* = 51)	TVD (*n* = 48)	*P*	Non-TVD (*n* = 69)	TVD (*n* = 31)	*P*
Clinical data						
Male, *n* (%)	28.0 (54.9%)	21.0 (43.8%)	0.267	47.0 (68.1%)	24 (77.4%)	0.343
Age, year	68.0 (60.0, 74.0)	68.5 (61.0, 77.3)	0.553	54.0 (47.0, 61.0)	55.0 (50.0, 64.0)	0.330
BMI, kg/m^2^	23.5 (21.6, 24.9)	23.9 (21.1, 26.6)	0.696	25.7 (23.9, 27.7)	26.2 (23.7, 28.4)	0.610
SBP, mmHg	135.0 (122.0, 153.0)	142.0 (130.0, 162.3)	0.252	140.0 (127.0, 150.0)	132.0 (124.0, 144.0)	0.271
DBP, mmHg	80.0 (74.0, 90.0)	82.5 (78.5, 90.0)	0.401	86.0 (80.0, 91.0)	82.0 (76.0, 88.0)	0.335
HR, BPM	76.0 (70, 80.0)	72.0 (68.0, 79.8)	0.260	70.0 (68.0, 78.5)	74.0 (70.0, 80.0)	0.475
Smoker, *n* (%)	11.0 (21.6%)	11.0 (22.9%)	0.872	33.0 (47.8%)	17.0 (54.8%)	0.517
Drinker, *n* (%)	4.0 (7.8%)	4.0 (8.3%)	0.929	11.0 (15.9%)	3.0 (9.7%)	0.404
PH, *n* (%)	37.0 (72.5%)	36.0 (75.0%)	0.782	44.0 (63.8%)	23.0 (74.2%)	0.305
T2DM, *n* (%)	16.0 (31.4%)	20.0 (41.7%)	0.287	19.0 (27.5%)	11.0 (35.5%)	0.422
Laboratory parameters						
PLT, 10^9^/L	201.0 (167.0, 250.0)	210.5 (174.3, 256.8)	0.747	227.0 (185.0, 271.5)	232.0 (191.0, 268.0)	0.849
WBC, 10^9^/L	6.6 (5.2, 7.6)	6.1 (5.2, 7.4)	0.739	7.0 (5.7, 8.8)	7.3 (6.2, 8.8)	0.531
ALT, U/L	20.0 (15,.0 31.0)	19.0 (14.0, 30.5)	0.443	28.0 (18.0, 39.5)	20.0 (15.0, 40.0)	0.285
AST, U/L	24.0 (20.0, 32.0)	23.0 (20.0, 29.0)	0.718	24.0 (19.0, 40.0)	23.0 (18.0, 32.0)	0.336
TBIL, μmol/L	13.8 (10.3, 18.0)	12.4 (10.3, 16.9)	0.372	13.7 (11.0, 16.6)	12.8 (10.2, 16.3)	0.498
eGFR, ml/min/1.73 m^2^	69.9 (59.6, 76.5)	63.0 (54.8, 76.4)	0.078	108.5 (97.9, 128.1)	106.4 (94.2, 123.7)	0.539
TC, mmol/L	6.0 (5.4, 6.7)	6.3 (5.2, 7.3)	0.317	5.6 (4.8, 6.6)	5.6 (4.5, 7.0)	0.890
TG, mmol/L	1.7 (1.3, 2.4)	1.7 (1.3, 2.2)	0.695	2.2 (1.4, 3.2)	1.7 (1.4, 2.6)	0.130
HDL-C, mmol/L	1.1 (1.0, 1.3)	1.2 (1.0, 1.3)	0.595	1.1 (1.0, 1.3)	1.0 (0.9, 1.1)	0.080
LDL-C, mmol/L	4.1 (3.4, 4.8)	4.7 (3.4, 5.2)	0.109	4.1 (3.2, 4.6)	4.0 (3.2, 4.9)	0.726
MLDL-C, mmol/L	5.1 (4.8, 5.9)	5.1 (4.8, 6.2)	0.634	4.7 (4.3, 5.4)	5.0 (4.5, 5.9)	0.209
With mutation in 3 FH major genes	14.0 (27.5%)	16.0 (33.3%)	0.524	12.0 (17.4%)	12 (38.7%)	0.021
With mutation in 11 FH related genes	22.0 (43.1%)	24.0 (50%)	0.494	31.0 (44.9%)	18 (58.1%)	0.224

FH, familial hypercholesterolemia; BMI, body mass index; SBP, systolic blood pressure; DBP, diastolic blood pressure; HR, heart rate; PH, primary hypertension; T2DM, type 2 diabetes mellitus; PLT, platelet; WBC, white blood cell; ALT, alanine transaminase; AST, aspartate aminotransferase; TBIL, total bilirubin; SUA, serum uric acid; eGFR, estimated glomerular filtration rate; TC, total cholesterol; TG, triglycerides; HDL-C, high-density lipoprotein cholesterol; LDL-C, low-density lipoprotein cholesterol; MLDL-C, modified LDL-C; CAD, coronary artery disease; TVD, three vessel disease.

### Polygenic risk scores including PCAD and controls

According to the age that patients suffering from CAD for the first time, CAD patients were devided into PCAD and Non-PCAD groups. Nagelkerke's *R*^2^ values derived from PRSice-2 for a range of single nucleotide polymorphism (SNP) among *P* value thresholds from 0.0011 to 1 in the PCAD base dataset, which were used to determine the best significance threshold for inclusion of SNPs required to distinguish between Non-PCAD and PCAD. There was a total of 146 SNPs found in base dataset. As the Supplementary Figure S1 showed, The largest Nagelkerke's *R*^2^ value generated was 0.31 suggesting the inclusion of 37 SNPs showed in Supplementary Table S2 at the *P* value threshold of 1.93 × 10^−8^ into the PRS model.

## Discussion

This study involved 223 individuals with MLDL-C levels ≥4.0 mmol/L who underwent CAG and FH screening via 11 genes. This study revealed that FH mutations were not a risk factor for the incidence of CAD among individuals with MLDL-C ≥ 4.0 mmol/L. However, it increases the severity of CAD, especially for individuals (MLDL-C ≥ 4.0 mmol/L) with relatively normal renal function. In addition, a total of 104 mutations were detected in this study, which might benefit FH patients with a previously missed diagnosis and provide appropriate lipid-lowering therapies.

As molecular technologies have improved, the importance of genetic testing for the clinical management and cardiovascular risk stratification of FH patients and their relatives has been confirmed (20–22). The genetic basis of FH is more complex than initially thought, and minor genes have gradually been identified. Laurens F Reeskamp et al. (23) reported that no pathogenic mutation was identified in the vast majority of patients undergoing genetic testing, and mutations in minor genes can provide an explanation. Another previous study suggested that a limited-variant screen has a significantly lower detection rate (8.4%) than a comprehensive diagnostic test (24). A child who was suspected as having FH with extremely elevated LDL-C levels was found to be caused by a pathogenic mutation in ABCG5 (25). In this study, we screened for causative variants of FH via an expanded gene panel via NGS. We found that FH was most common in individuals with MLDL-C levels between 6 and 6.99 mmol/L. This result will help us to genetically identify FH more efficiently.

In total, 104 mutations, including 44 in major genes and 60 in minor genes, were detected via NGS. As expected, LDLR was the most common gene causing FH, accounting for 69.5% of the cases in the 3-gene panel. According to the Global Variome Shared LOVD updated on February 16, 2024 (https://databases.lovd.nl/shared/genes/LDLR), approximately 4,073 variants were reported in the LDLR gene. However, variants in the PCSK9 gene were not common in this study, which was similar to the findings of a previous study conducted in Taiwan (26).

As stated above, FH is caused by any abnormal expression of genes resulting in disorders of LDL-C metabolism. Patients with increased LDL-C levels are most likely missed because of rare or unknown FH mutations. A recent meta-analysis involving 18 studies revealed that the relative risk of cardiovascular events and death in the general population with FH was 2.85 (27). However, whether individuals with FH had more serious CVD than did those with non-FH among individuals with high LDL-C levels resulting from other inherited diseases or secondary factors is not fully clear.

This study revealed that there was no significant difference in the incidence of FH mutation between the CAD group and the non-CAD group in individuals who underwent CAG with MLDL-C ≥ 4.0 mmol/L. However, the CAD group had higher WBC counts than the non-CAD group did (*P* < 0.05). This outcome was similar to that of a previous study indicating that a increase in inflammatory risk increases the vascular injury rate (28).

Among the CAD group, we found that TVD patients had a greater proportion of patients with mutations in 3 FH major genes than non-TVD patients did. However, multivariate logistic regression analyses revealed that only the eGFR was an independent risk factor for TVD. As MJ Sarnak et al. (29) reported, CVD is the leading cause of morbidity and mortality among patients with chronic kidney disease. As the eGFR decreases below 60–75 ml/min/1.73 m^2^, the probability of developing CAD increases linearly. This study also indicated that chronic kidney disease was a major risk factor for CAD because of inflammation, oxidative stress, and abnormal calcium‒phosphorus metabolism (29). To eliminate the impact of renal function, subgroup analysis was conducted and further revealed that having mutations in 3 FH major genes was an independent risk factor for TVD among CAD patients with high eGFRs (≥87.4 ml/min/1.73 m^2^). Figure 3 shows that TVD was more common in the FH with both major and minor gene groups than in the non-FH group. Considering their earlier LDL-C accumulation, coronary artery stenosis in FH patients might be more severe. In addition, a previous study suggested that numerous variants in genes have cumulative effects on protein translation, structure, and function (30). Is there a cumulative effect of variants in minor genes and major genes on the clinical phenotype? More analyses are needed to answer this question.

This study also explored the association between PRS and PCAD. When including 37 SNPs, there was a significant difference of PRSs between PCAD and CAD patients (*R*^2^ = 0.31). At present, personalized medicine and precision medicine were increasingly more popular in clinical practice. With the advent of PRS, preventive intervention of CAD can be adopted in early stage according to assessing human genome information after birth. A previous review published at JACC Asia reported that PRS have generally been shown to offer incremental information for risk prediction of CAD beyond the use of traditional risk factors (31). AV Khera et al. (32). found that high polygenic score was associated with a 3.7-fold (95% CI: 3.1–4.6; *P* < 0.001) increased odds of early-onset myocardial infarction. And high polygenic score had a 10-fold higher prevalence than monogenic FH among patients presents with early-onset myocardial infarction. In additon, another study also identified that CAD can be predicted by using PRS, comprising common SNVs associated with CAD risk (33).

Further genetic testing is needed to screen for FH, including variants in minor genes, among individuals with high LDL-C levels. Genetic testing could improve screening for FH, and play an important role in the early diagnosis of FH to prevent serious complications. Furthermore, genetic testing could contribute to the stratification of severe cases and identify appropriate drug therapies. Patients with FH mutations in both major and minor genes might need more severe therapeutic regimens.

## Strengths

This study had several strengths. First, all of the subjects in this study with an MLDL-C level ≥4.0 mmol/L underwent CAG. Second, this study used targeted next-generation sequencing of all the coding exons of the 3 major genes and 8 minor genes and revealed a total of 104 FH mutations. In addition, this study performed a subgroup analysis to further explore the impacts of FH mutation on the severity of CAD.

## Limitations

Some limitations must be considered. First, owing to the retrospective and cross-sectional design, some blood samples were lost, and clinical data, such as LDL-C levels before lipid-lowering treatment, still exhibited discrepancies compared with real values even though the data were adjusted carefully. Second, as a retrospective study, this study did not perform a functional analysis to determine the pathogenicity of the detected variants. In the future, prospective and cohort studies should be conducted for further analysis.

## Conclusion

FH mutation did not increase the rate of CAD among those with an MLDL-C level ≥4.0 mmol/L. However, among CAD patients (MLDL-C level ≥4.0 mmol/L) with almost normal renal function, the probability of TVD in those with FH mutations in 3 major genes is 3.00 times greater than that in those without FH mutations. Furthermore, PRS had an ability to offer information to predict the PCAD beyond the traditional risk factors.

## Data Availability

The original contributions presented in the study are publicly available. This data can be found here: https://www.ncbi.nlm.nih.gov/bioproject/PRJNA1174075.

## Supplementary material

Supplementary Figure S1 The result of Polygenic risk scores to predict PCAD.
