# Supplementary material for: Cardiovascular disease risk in patients with elevated LDL-C levels: FH vs. non-FH
Source: Front Cardiovasc Med. 2024 Oct 24;11:1434392. doi: 10.3389/fcvm.2024.1434392 (PMC11540629; doi:10.3389/fcvm.2024.1434392)
Supplement: Supplementary file 1 [file Table1.pdf]

Supplementary Table 1 Whole variants identified by genetic testing

Gene	Transcript	Exon	Nucleotide change	Effect on protein	Pathogenicity	SNP ID	Function	Number of patients
LDLR	NM_000527	exon2	c.97C>T	p.Q33X	Pathogenic	rs121908024	Stop gain	1
LDLR	NM_000527	exon3	c.211G>A	p.G71R	Likely benign	rs766903209	Nonsynonymous	1
LDLR	NM_000527	exon3	c.268G>A	p.D90N	Likely pathogenic	rs749038326	Nonsynonymous	3
LDLR	NM_000527	exon3	c.313+1G>A	NA	Pathogenic	rs112029328	NA	1
LDLR	NM_001195799	exon3	c.221G>A	p.R74H	Uncertain significance	rs201102461	Nonsynonymous	3
LDLR	NM_001195799	exon3	c.476T>G	p.F159C	Uncertain significance	rs879254586	Nonsynonymous	1
LDLR	NM_001195799	exon3	c.558C>A	p.D186E	Likely pathogenic	rs121908028	Nonsynonymous	1
LDLR	NM_001195799	exon4	c.646C>T	p.R216W	Uncertain significance	rs200990725	Nonsynonymous	2
LDLR	NM_001195799	exon4	c.688G>A	p.V230I	Uncertain significance	rs749220643	Nonsynonymous	2
LDLR	NM_001195800	exon4	c.354C>A	p.S118R	Likely pathogenic	rs140241383	Nonsynonymous	1
LDLR	NM_001195800	exon5	c.544C>T	p.R182X	Pathogenic	rs769737896	Stop gain	1
LDLR	NM_001195800	exon6	c.625T>G	p.C209G	Likely pathogenic	rs773064328	Nonsynonymous	1
LDLR	NM_001195800	exon7	c.691G>A	p.A231T	Likely pathogenic	rs730882099	Nonsynonymous	1

LDLR	NM_001195800	exon7	c.743G>A	p.R248Q	Likely pathogenic	rs773658037	Nonsynonymous	1
LDLR	NM_001195800	exon7	c.764T>C	p.I255T	Likely pathogenic	rs879254849	Nonsynonymous	1
LDLR	NM_001195800	exon9	c.1095G>A	p.W365X	Pathogenic	rs879254952	Stop gain	1
LDLR	NM_001195800	exon9	c.1187A>G	p.N396S	Likely pathogenic	rs758194385	Nonsynonymous	1
LDLR	NM_001195800	exon10	c.1243C>T	p.H415Y	Uncertain significance	rs730882109	Nonsynonymous	3
LDLR	NM_001195800	exon10	c.1261G>A	p.D421N	Uncertain significance	rs201971888	Nonsynonymous	3
LDLR	NM_001195800	exon10	c.1279C>T	p.R427W	Uncertain significance	rs373371572	Nonsynonymous	2
LDLR	NM_001195800	exon10	c.1280G>A	p.R427Q	Likely pathogenic	rs201102492	Nonsynonymous	2
LDLR	NM_001195800	exon10	c.1292T>C	p.L431S	Likely pathogenic	rs879255025	Nonsynonymous	1
LDLR	NM_001195800	exon11	c.1375G>A	p.A459T	Likely pathogenic	rs879255066	Nonsynonymous	5
LDLR	NM_001195800	exon11	c.1438T>C	p.S480P	Likely pathogenic	rs879255079	Nonsynonymous	1
LDLR	NM_000527	exon14	c.1988-8T>A	NA	Uncertain significance	NA	NA	1
LDLR	NM_001195800	exon12	c.1498G>T	p.E500X	Pathogenic	NA	Stop gain	1
LDLR	NM_001195800	exon12	c.1499A>T	p.E500V	Likely pathogenic	NA	Nonsynonymous	1
LDLR	NM_001195800	exon12	c.1542delC	p.L514fs	Likely pathogenic	NA	Frameshift deletion	1
APOB	NM_000384	exon26	c.11456A>C	p.Q3819P	Uncertain significance	NA	Nonsynonymous	1

APOB	NM_000384	exon26	c.10579C>T	p.R3527W	Uncertain significance	rs144467873	Nonsynonymous	5
APOB	NM_000384	exon26	c.10294C>G	p.Q3432E	Likely benign	rs1042023	Nonsynonymous	1
APOB	NM_000384	exon26	c.9191T>G	p.L3064X	Pathogenic	NA	Stop gain	3
APOB	NM_000384	exon26	c.7223C>T	p.S2408F	Uncertain significance	rs140027955	Nonsynonymous	1
APOB	NM_000384	exon26	c.6898C>G	p.Q2300E	Uncertain significance	NA	Nonsynonymous	1
APOB	NM_000384	exon26	c.6551A>G	p.Y2184C	Uncertain significance	rs184512808	Nonsynonymous	2
APOB	NM_000384	exon26	c.6455C>T	p.T2152I	Uncertain significance	rs752858245	Nonsynonymous	1
APOB	NM_000384	exon25	c.4111G>A	p.A1371T	Uncertain significance	rs780170292	Nonsynonymous	1
APOB	NM_000384	exon23	c.3607A>G	p.S1203G	Uncertain significance	rs78875649	Nonsynonymous	1
APOB	NM_000384	exon22	c.3404G>A	p.S1135N	Uncertain significance	rs778519921	Nonsynonymous	1
APOB	NM_000384	exon15	c.2204T>C	p.V735A	Uncertain significance	NA	Nonsynonymous	1
APOB	NM_000384	exon14	c.1934T>G	p.L645R	Uncertain significance	NA	Nonsynonymous	1
APOB	NM_000384	exon4	c.288G>T	p.Q96H	Uncertain significance	rs186544754	Nonsynonymous	1
PCSK9	NM_174936	exon1	c.43_48del	p.15_16del	Uncertain significance	rs778382130	Nonsynonymous	1
PCSK9	NM_174936	exon3	c.499C>T	p.R167W	Uncertain significance	rs137878146	Nonsynonymous	1
APOBR	NM_018690	exon2	c.67G>A	p.G23S	Uncertain significance	rs577322366	Nonsynonymous	1
APOBR	NM_018690	exon2	c.115C>T	p.R39W	Uncertain significance	rs751358620	Nonsynonymous	1

APOBR	NM_018690	exon2	c.235G>A	p.G79R	Uncertain significance	rs867103469	Nonsynonymous	1
APOBR	NM_018690	exon2	c.1697T>C	p.L566P	Uncertain significance	NA	Nonsynonymous	1
APOBR	NM_018690	exon2	c.1811C>T	p.A604V	Uncertain significance	NA	Nonsynonymous	1
APOBR	NM_018690	exon2	c.2345T>C	p.L782S	Uncertain significance	NA	Nonsynonymous	1
APOBR	NM_018690	exon2	c.2419C>G	p.Q807E	Uncertain significance	NA	Nonsynonymous	1
APOBR	NM_018690	exon3	c.3193G>A	p.V1065M	Uncertain significance	NA	Nonsynonymous	1
APOBR	NM_018690	exon3	c.3199G>A	p.A1067T	Uncertain significance	NA	Nonsynonymous	1
LIPA	NM_001288979	exon5	c.446G>A	p.C149Y	Uncertain significance	NA	Nonsynonymous	1
LIPA	NM_001288979	exon2	c.59A>C	p.Q20P	Uncertain significance	NA	Nonsynonymous	8
LPA	NM_005577	exon39	c.6113G>T	p.R2038I	Likely benign	NA	Nonsynonymous	1
LPA	NM_005577	exon37	c.5831delG	p.G1944fs	Likely pathogenic	NA	Frameshift deletion	1
LPA	NM_005577	exon35	c.5550A>C	p.L1850F	Uncertain significance	NA	Nonsynonymous	1
LPA	NM_005577	exon34	c.5438C>T	p.P1813L	Uncertain significance	rs771057576	Nonsynonymous	1
LPA	NM_005577	exon32	c.5185C>T	p.R1729W	Uncertain significance	rs374776600	Nonsynonymous	1
LPA	NM_005577	exon31	c.5085_5100del	p.D1695fs	Likely pathogenic	NA	Frameshift deletion	1

LPA	NM_005577	exon30	c.4973+1G>A	NA	Pathogenic	rs201888157	NA	18
LPA	NM_005577	exon28	c.4520G>A	p.G1507E	Uncertain significance	rs769200592	Nonsynonymous	1
LPA	NM_005577	exon26	c.4289+1G>T	NA	Uncertain significance	rs41272114	NA	1
LPA	NM_005577	exon26	c.4257G>T	p.W1419C	Likely benign	NA	Nonsynonymous	1
LPA	NM_005577	exon26	c.4240T>C	p.S1414P	Likely benign	rs770419679	Nonsynonymous	1
LPA	NM_005577	exon26	c.4236G>A	p.W1412X	Pathogenic	NA	Stop gain	1
LPA	NM_005577	exon25	c.4046C>T	p.T1349M	Likely benign	rs201200716	Nonsynonymous	1
LPA	NM_005577	exon24	c.3919C>T	p.Q1307X	Likely pathogenic	NA	Nonsynonymous	1
LPA	NM_005577	exon21	c.3319G>T	p.D1107Y	Uncertain significance	NA	Nonsynonymous	1
LPA	NM_005577	exon16	c.2503C>T	p.R835X	Pathogenic	rs746733669	Stop gain	1
LPA	NM_005577	exon7	c.1051A>G	p.S351G	Likely benign	NA	Nonsynonymous	1
LRPAP1	NM_002337	exon7	c.989G>A	p.R330Q	Likely benign	rs143714310	Nonsynonymous	1
LRPAP1	NM_002337	exon7	c.884A>C	p.Q295P	Uncertain significance	NA	Nonsynonymous	1
LRPAP1	NM_002337	exon6	c.808A>C	p.T270P	Uncertain significance	NA	Nonsynonymous	4
LRPAP1	NM_002337	exon5	c.710G>A	p.R237H	Uncertain significance	rs760183295	Nonsynonymous	2
LRPAP1	NM_002337	exon2	c.220G>A	p.V74M	Likely benign	rs573489074	Nonsynonymous	1
LDLRAP1	NM_015627	exon2	c.167C>T	p.T56M	Uncertain significance	rs752849346	Nonsynonymous	1

LDLRAP1	NM_015627	exon5	c.517C>T	p.Q173X	Pathogenic	rs866025690	Stop gain	1
STAP1	NM_001317769	exon1	c.19C>T	p.P7S	Uncertain significance	NA	Nonsynonymous	1
STAP1	NM_001317769	exon6	c.554A>G	p.K185R	Likely pathogenic	NA	Nonsynonymous	1
ABCG5	NM_022436	exon13	c.1904T>A	p.V635D	Uncertain significance	NA	Nonsynonymous	1
ABCG5	NM_022436	exon13	c.1901T>G	p.L634R	Uncertain significance	NA	Nonsynonymous	1
ABCG5	NM_022436	exon12	c.1762+1G>A	NA	Pathogenic	rs754944896	NA	1
ABCG5	NM_022436	exon11	c.1528C>A	p.H510N	Uncertain significance	rs199984328	Nonsynonymous	2
ABCG5	NM_022436	exon10	c.1337G>A	p.R446Q	Uncertain significance	rs536081800	Nonsynonymous	4
ABCG5	NM_022436	exon10	c.1336C>T	p.R446X	Pathogenic	rs199689137	Stop gain	2
ABCG5	NM_022436	exon10	c.1325-2A>G	NA	Pathogenic	NA	NA	1
ABCG5	NM_022436	exon8	c.1067T>A	p.F356Y	Uncertain significance	rs775348848	Nonsynonymous	1
ABCG5	NM_022436	exon7	c.838A>G	p.M280V	Uncertain significance	NA	Nonsynonymous	1
ABCG5	NM_022436	exon6	c.751C>T	p.Q251X	Pathogenic	rs140111105	Stop gain	1
ABCG5	NM_022436	exon6	c.668T>C	p.L223P	Uncertain significance	rs186904986	Nonsynonymous	1
ABCG5	NM_022436	exon6	c.662C>T	p.T221I	Uncertain significance	NA	Nonsynonymous	1
ABCG5	NM_022436	exon5	c.610G>A	p.A204T	Uncertain significance	rs765266332	Nonsynonymous	1
ABCG5	NM_022436	exon4	c.438G>C	p.E146D	Uncertain significance	rs748096191	Nonsynonymous	1

ABCG8	NM_001357321	exon5	c.644G>T	p.G215V	Uncertain significance	rs769781582	Nonsynonymous	1
ABCG8	NM_022437	exon5	c.694+5G>C	NA	Uncertain significance	rs557890655	NA	2
ABCG8	NM_001357321	exon6	c.786C>A	p.N262K	Uncertain significance	rs199737442	Nonsynonymous	1
ABCG8	NM_001357321	exon9	c.1253T>A	p.I418N	Uncertain significance	rs201659189	Nonsynonymous	1
ABCG8	NM_001357321	exon9	c.1282A>G	p.M428V	Uncertain significance	rs147194762	Nonsynonymous	2
ABCG8	NM_001357321	exon9	c.1348C>G	p.L450V	Uncertain significance	NA	Nonsynonymous	1
ABCG8	NM_001357321	exon11	c.1492G>A	p.G498R	Uncertain significance	rs368551459	Nonsynonymous	1
ABCG8	NM_001357321	exon11	c.1622G>A	p.C541Y	Uncertain significance	NA	Nonsynonymous	2
ABCG8	NM_001357321	exon12	c.1874G>T	p.G625V	Uncertain significance	rs763289865	Nonsynonymous	1

NA not available
