# Supplementary material for: Cardiovascular disease risk in patients with elevated LDL-C levels: FH vs. non-FH
Source: Front Cardiovasc Med. 2024 Oct 24;11:1434392. doi: 10.3389/fcvm.2024.1434392 (PMC11540629; doi:10.3389/fcvm.2024.1434392)
Supplement: Supplementary file 2 [file Table2.pdf]

Supplementary Table 2 A total of 37 SNPs included in polygenic risk scores model

CHR	SNP	BP	OR	*P*
2	rs4148195	43812494	0.3601	0.001055
2	rs1041968	21009932	3.216	0.03198
1	rs2483205	55052643	0.6199	0.06649
2	rs2954805	43882019	1.588	0.07868
19	rs72658879	11133332	0.5823	0.09289
6	rs3798220	160540105	1.978	0.09998
4	rs1741555	3511478	0.6823	0.1016
2	rs13306198	21037212	2.125	0.1176
16	rs40833	28498723	0.71	0.1228
16	rs149271	28495551	0.5932	0.1787
2	rs2954801	43878918	0.7684	0.2115
19	rs10423288	11105170	0.6823	0.2259
19	rs2738464	11131631	0.7167	0.2375
2	rs1367117	21041028	1.39	0.2657
6	rs3124784	160531806	1.392	0.2687
22	rs74445350	43891749	1.278	0.3341
6	rs9365196	160635080	1.381	0.344
4	rs3816821	67593219	1.221	0.3671
10	rs1051338	89247603	1.246	0.3823
19	rs3745678	11100403	0.8001	0.4689
4	rs2242330	67581531	0.8051	0.4709
1	rs6687605	25563141	1.136	0.5512
1	rs2479410	55040188	0.9619	0.917
2	rs1042031	21002881	0.7515	0.5399
2	rs2163204	21008515	0.8389	0.7124
2	rs13306194	21029662	0.9173	0.8229
2	rs4148211	43844604	1.247	0.505
2	rs138296512	43874398	1.111	0.6441
2	rs11124951	43880670	0.916	0.7724
4	rs1794441	3514642	0.8647	0.691
4	rs1799746	3520027	1.22	0.6069
4	rs11556614	67577250	1.026	0.9164
10	rs2297472	89225233	0.8794	0.6159
10	rs2071509	89228493	1.063	0.7683
19	rs688	11116926	1.143	0.6418
19	rs13306501	11128132	0.8965	0.6985
22	rs2017461	43889885	0.7364	0.5328

CHR chromosome, SNP single nucleotide polymorphism, BP base pair, OR odds ratio
