# Supplementary figures and images for: Cardiovascular disease risk in patients with elevated LDL-C levels: FH vs. non-FH
Source: Front Cardiovasc Med. 2024 Oct 24;11:1434392. doi: 10.3389/fcvm.2024.1434392 (PMC11540629; doi:10.3389/fcvm.2024.1434392)

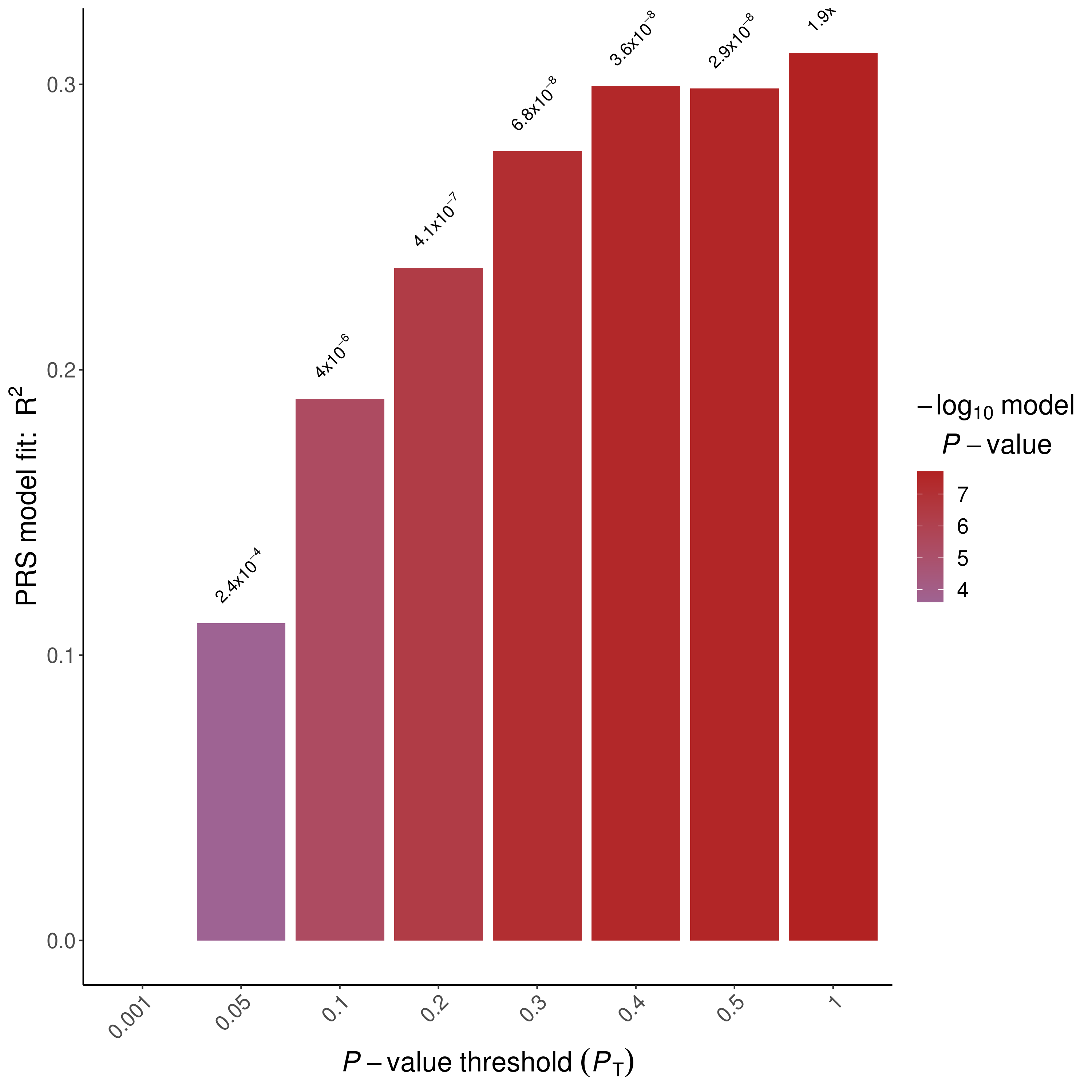

Supplement: Supplementary Figure S1 — The result of Polygenic risk scores to predict PCAD. [file Image1.tif]
